# Protocol for generating and using human iPSC-derived microglia-containing air-liquid-interface cortical organoid cultures

**DOI:** 10.1016/j.xpro.2025.103915

**Published:** 2025-06-30

**Authors:** Marta Cañizares Luna, Mayte Mars, Channa E. Jakobs, Christiaan F.M. Huffels, Arthur Ermakov, Elly M. Hol, R. Jeroen Pasterkamp

**Affiliations:** 1 Department of Translational Neuroscience, UMC Utrecht Brain Center, University Medical Center Utrecht, Utrecht University, Utrecht 3584 CG, the Netherlands

**Keywords:** Cell Biology, Cell culture, Cell isolation, Immunology, Molecular Biology, Neuroscience, Stem Cells, Cell Differentiation, Organoids

## Abstract

Here, we present a protocol for generating long-term microglia-containing air-liquid-interface cortical organoid (MG-ALI-CO) cultures. This approach minimizes necrotic core formation, a common limitation of extended organoid cultures, favoring microglia survival and homeostasis. We describe steps for generating air-liquid-interface cortical organoids (ALI-COs), integrating macrophage precursors, and maintaining MG-ALI-COs. Additionally, we outline several experimental analyses of MG-ALI-COs, including immunostaining, imaging, and patch-clamp electrophysiological recordings. This model provides a physiologically relevant system to investigate human neuroimmune interactions in a 3D brain-like environment.

## Highlights

• Steps for generating iPSC-derived MacPre and ALI-COs
• Instructions for integrating MacPre into ALI-CO and MG-ALI-CO maintenance
• Guidance on how to use MG-ALI-COs for studying neuroimmune interactions

## Abstract (editor highlights)

Publisher’s note: Undertaking any experimental protocol requires adherence to local institutional guidelines for laboratory safety and ethics.

## Graphical abstract

**Figure:**
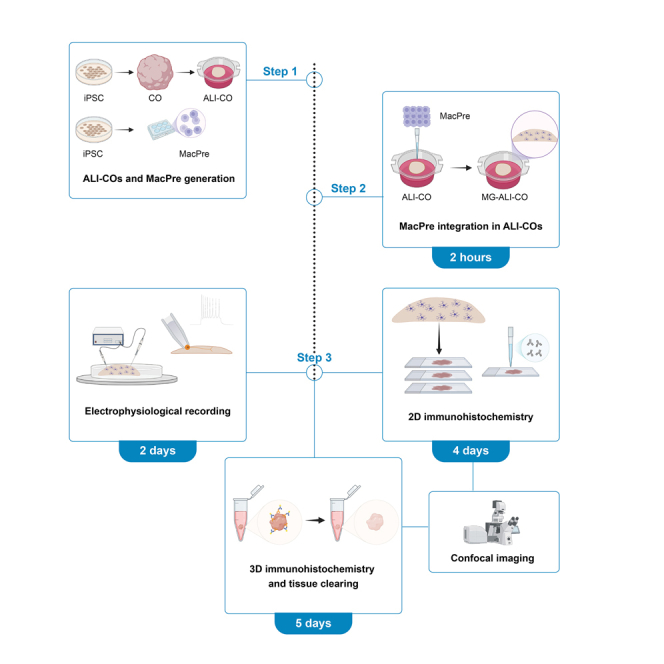

## Before you begin

Microglia are the resident immune cells of the central nervous system (CNS) and are implicated in numerous neurological disorders.^1^^,^^2^ Rodent models have been essential for understanding key aspects of microglia biology, but differences between rodent and human microglia^3^ highlight the need for additional models to fully understand human pathology. Induced pluripotent stem cell (iPSC)-derived microglia^4^ have greatly facilitated studies on human microglia in the past several years. 2-dimensional (2D) systems have been used successfully to dissect disease mechanisms,^5^ but they do not provide the 3D, multicellular environment of the human brain. Therefore, distinct protocols have been developed to incorporate microglia into neural organoids,^6^^,^^7^^,^^8^ which provide a more physiologically relevant environment and allow the study of neuroimmune interactions.^9^

Necrotic core formation occurs in long-term neural organoid cultures due to reduced nutrient and oxygen supply, negatively affecting microglia survival.^10^ Recent advances offer the possibility of generating organotypic slices from neural organoids, so called air-liquid-interface cerebral organoids (ALI-COs^11^). This approach enables extended culturing periods by facilitating sufficient oxygen and nutrient availability, which promotes increased neuronal maturation and minimizes necrotic core formation.^11^ Here, we present a comprehensive step-by-step protocol on how to generate and use microglia-containing cortical ALI-COs (MG-ALI-COs) in different experimental settings. Overall, MG-ALI-COs provide a physiologically relevant human *in vitro* system that allows long-term microglia co-culture, enables tight control of microglia number or genetic background, and is characterized by highly ramified and evenly spaced microglia. The experimental applications described – 2D and 3D immunohistochemistry (IHC), confocal imaging and patch clamp electrophysiology – can be applied to ALI-COs both with and without microglia, making the protocol relevant to a large group of researchers.

***Note:*** In this protocol we use the abbreviation “ALI-CO” as was done in the original study.^11^ However, whereas the study of Giandomenico and colleagues was based on cerebral organoids,^12^ here cortical organoids^13^ are used.

### Institutional permission

Approval for the generation and use of iPSC lines used in this protocol was granted by the Medical Ethics Committee (METC) of the University Medical Center Utrecht. Informed consent was obtained from all donors. Readers that intend to use this protocol need to obtain permission from their relevant institutions.

### Induced pluripotent stem cell culturing

**Timing: variable**

Culture iPSCs in StemFlex on Geltrex-coated dishes, performing a complete medium change three times per week. Once the iPSCs have reached 80 to 90% confluency, split with 0.5 mM EDTA and culture for 24 hours in StemFlex, supplemented with 5 μM Y-27632 dihydrochloride, before switching back to StemFlex.

**CRITICAL:** Before starting any differentiation protocol, iPSCs should be 70–90% confluent and stem cell colonies should have no signs of mis-differentiation (e.g., non-compact colonies).

### Cortical organoid generation

**Timing: ∼50 days**

This section provides the steps needed to generate cortical organoids, adapted from *Yoon et al., 2019*.^13^

1. On day in vitro 0 (DIV0) of the organoid culture, seed 9000 iPSCs in 150 μL hES0 medium, supplemented with 50 μM Y-27632 dihydrochloride and 4 ng/mL FGF2, per well of an ultra-low attachment (ULA) 96-well plate.
2. Culture organoids until approximately DIV50 in the ULA 96-well plate.
  a. On DIV2 and DIV4, change all medium to 150 μL hES0 medium, supplemented with 2.5 μM Dorsomorphin and 10 μM SB-431542.
  b. On DIV6, switch completely to neural medium, supplemented with 20 ng/mL EGF and 20 ng/mL FGF2.
  c. Perform a complete medium change three times per week.
  d. On DIV25, replace all medium with neural medium, supplemented with 20 ng/mL BDNF and 20 ng/mL NT-3.
  e. Replace all medium three times a week.
  f. After DIV43, change medium completely three times per week with neural medium without supplements until ALI-CO slicing.

### Macrophage precursor differentiation

**Timing: 3–8 weeks**

This section describes the protocol to generate iPSC-derived macrophage precursors (MacPre), adapted from Haenseler et al., 2017.^14^ The steps in this section should be performed in parallel with the steps in “cortical organoid generation” and “air-liquid-interface cortical organoid generation”.

3. On DIV0, generate yolk-sac embryoid bodies (YS-EBs) in an ULA 96-well plate. Per well, seed 9000 iPSCs in 150 μL SF-EB medium supplemented with 50 μM Y-27632, 50 ng/mL BMP-4, 20 ng/mL SCF and 50 ng/mL VEGF.
4. On DIV2, refresh all medium containing all the abovementioned factors.
5. On DIV4, harvest YS-EBs and wash in 1X PBS before transferring 10 to 15 YS-EBs per well of a 6 well-plate in MacPre medium, supplemented with 100 ng/mL M-CSF and 25 ng/mL IL-3.
6. Replace 2/3^rd^ of the MacPre medium every 5 to 7 days. Be careful to not aspirate YS-EBs.
7. MacPre start to appear in the medium between 2 to 3 weeks after YS-EB transfer.

***Note:*** YS-EBs will develop cystic yolk-sac-like structures and often adhere to the bottom of the well.

### Air-liquid-interface cortical organoid generation

**Timing: ∼90 days**

This section describes the protocol to slice cortical organoids around DIV50, adapted from Giandomenico et al., 2019^11^ and Giandomenico et al., 2020.^15^

***Note:*** Here, organoid slicing is performed soon after ending factor supplementation in the organoid medium to prevent necrotic core formation. Slicing at later time points is also possible, as shown by Giandomenico et al., 2019.^11^

**Figure 1:**
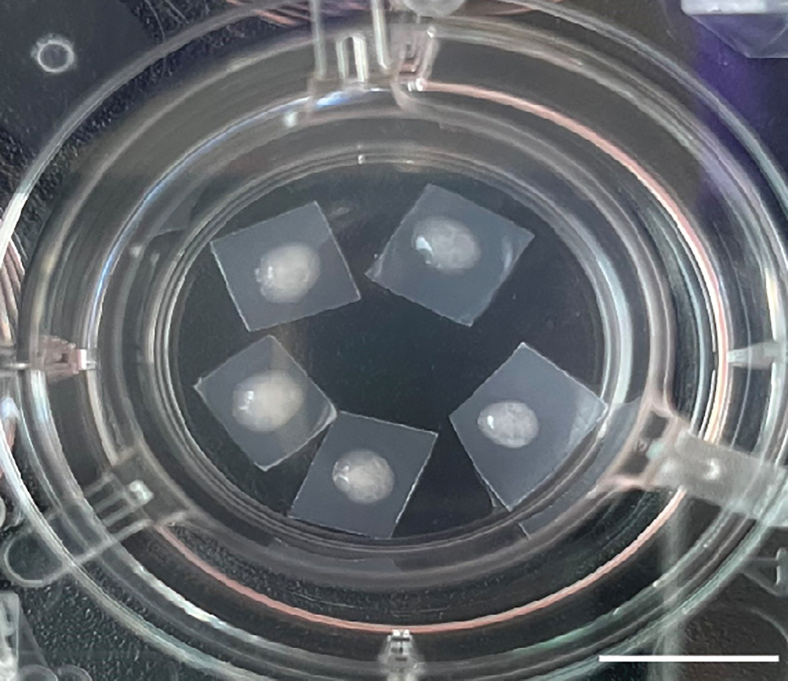
ALI-COs on membranes and cell culture insert Individual DIV121 ALI-COs (63 days after slicing) on 0.45 μm membranes in a hanging cell culture insert in a 6 well-plate. Top view. Scale bar, 1 mm.

8. Prepare the slicing setup.
  a. Profusely spray the vibrating microtome with 70% ethanol, place in a flow hood and expose to UV light for 30 minutes to sterilize.
    **CRITICAL:** Ensure that especially the parts of the vibratome which are in contact with the organoids, such as the blade, mounting plate and tray, are exposed to UV light and sprayed with 70% ethanol.
  b. Prepare 3% low-melting agarose solution in 1X HBSS.
    ***Note:*** The agarose can be prepared in advance. Place the agarose during the slicing process on a shaker (60 rpm) at 50°C to keep it in a liquid state for the embedding.
  c. Expose 0.45 μm membranes on both sides to UV light for 30 minutes for sterilization and cut into ∼5 × 5 mm pieces with sterile scissors.
  d. Place 1X HBSS in the freezer to obtain a slushy, but not completely frozen, solution.
9. Embed up to 15 organoids in 3% low-melting agarose.
  a. Collect DIV50 organoids with a P1000 pipette with a wide bore tip and wash them quickly in a 6 cm petri dish with 1X HBSS at 18 to 20°C.
    **CRITICAL:** Collect well-developed, round organoids for slicing. Organoids that are too small (<1 mm diameter) or have too many buds (*i.e.,* typical signs of miss differentiation) will not survive the slicing procedure.
  b. Transfer the organoids to a 35 mm or 6 cm petri dish, depending on how many organoids are embedded, filled with melted agarose and move organoids around with a P20 tip.
    ***Note:*** Use a 6 cm petri dish when embedding more than 6 (up to 15) organoids.
    **CRITICAL:** To avoid loosening of the organoid from the agarose during the slicing, it is important to mix the HBSS that was transferred with the organoid with the surrounding agarose by moving around the organoids with a P20 tip.
  c. Ensure that organoids are placed in the same horizontal plane within the agarose by letting them sink to the bottom of the dish.
    **CRITICAL:** Make sure that the agarose solution does not contain bubbles when using it for embedding. Bubbles might appear after preparation but will diminish quickly afterward.
  d. Place embedded organoids on an ice block for ∼20 minutes to let the agarose polymerize.
10. Mount the agarose block containing the embedded organoids on a vibrating microtome.
  a. Remove excess agarose around the organoids with a blade and lift the embedded organoids out of the petri dish.
  b. To avoid unnecessary slicing of agarose not containing organoids, invert the agarose block so that the organoids are in the upper part of the agarose block.
  c. Glue the inverted agarose block with embedded organoids to the holder of the vibrating microtome.
11. Slice organoids with the vibrating microtome.
  a. Fill the vibratome chamber with ice-cold 1X HBSS. Change the 1X HBSS between organoids from different iPSC lines.
  b. Cut organoids into 250 μm thick sections (speed 0.12 mm/s, frequency 45 Hz).
  c. Collect sections with a P1000 pipette with a wide bore tip and transfer the sections into a 6-well plate with prewarmed recovery medium with freshly added penicillin-streptomycin-amphotericin (PSA, final concentration 1X).
  d. Incubate the sections in the 6-well plate with recovery medium for at least one hour at 37°C.
12. Transfer ALI-COs to the culturing insert and continue culture until harvesting (Figure 1).
  a. Prepare a 6-well plate with 1.5 mL ALI-CO medium with freshly added PSA and one hanging cell culture insert per well.
  b. Place 4 to 6 cut membranes in the hanging cell culture insert.
    ***Note:*** 0.45 μm membranes are used to make harvesting of ALI-COs easier. By using individual membranes instead of directly culturing ALI-COs on the inserts, it is possible to easily collect single ALI-COs. Sterile tweezers can be used to carefully lift the membrane and collect specific ALI-COs.
  c. Transfer the ALI-COs to the membranes in the pre-warmed 6-well plate using a P1000 pipette with a wide bore tip. Place one ALI-CO per membrane. Place the ALI-CO in the middle of the membrane (as shown in Figure 1).
  d. Refresh the ALI-CO medium completely with medium containing freshly added PSA the following day.
  e. Subsequently, perform a complete medium change three times per week until harvesting.

## Key resources table

**Table:** 

REAGENT or RESOURCE	SOURCE	IDENTIFIER
**Antibodies**		

CTIP2 (1:500)	Abcam	#AB18465, RRID: AB_2064130
SATB2 (1:200)	Abcam	#AB34735, RRID: AB_2301417
FOXG1 (1:200)	Abcam	#AB18259, RRID: AB_732415
PSD-95 (1:400)	NanoTag Biotechnologies	#N3702-AF568-L, RRID: AB_3076103
SYP (1:500)	Synaptic Systems	#101 308, RRID: AB_2924959
IBA1 (1:1,000)	Wako	#019-19741, RRID: AB_839504
PU.1 (1:1,000)	Cell Signaling	#2258, RRID: AB_2186909
CD68 (1:100)	Dako	#M0814, RRID: AB_2314148
MAP2 (1:1,000)	Abcam	#AB92434, RRID: AB_2138147
Donkey-anti-guinea pig AF488	Jackson ImmunoResearch	#706-545-148, RRID: AB_2340472
Donkey-anti-mouse AF568	Thermo Fisher Scientific	#A10037, RRID: AB_11180865
Donkey-anti-chicken AF647	Jackson ImmunoResearch	#703-605-155, RRID: AB_2340379
Donkey-anti-rat AF568	Abcam	#AB150154, RRID: AB_2813834
Donkey anti-rabbit AF488	Invitrogen	#A21206, RRID: AB_2535792

**Chemicals, peptides, and recombinant proteins**		

StemFlex	Gibco	#A3349401
Geltrex	Gibco	#A1413202
EDTA	Invitrogen	#15-575-020
Y 27632 dihydrochloride	Axon Medchem	#1683
FGF2	PeproTech	#100-18B
KOSR	Gibco	#10828028
FBS	Sigma-Aldrich	#F7524
L-glutamine	Gibco	#25030024
MEM-NEAA	Gibco	#11140035
2-mercaptoethanol	Fisher Scientific	#11441711
DMEM/F-12	Gibco	#11320074
Penicillin-streptomycin	Gibco	#15140122
Neurobasal medium	Gibco	#21103049
B27 without vitamin A	Gibco	#12587010
Recombinant human EGF	R&D Systems	#236-EG
Dorsomorphin	R&D Systems	#3093
SB-431542	Axon Medchem	#AXON 1661
BDNF	STEMCELL Technologies	#78005.1
NT-3	PreproTech	#450-03B
Low-melting agarose	Fisher Bioreagents	#BP165-25
10X HBSS, calcium, magnesium	Gibco	#14065049
B27 with vitamin A	Gibco	#A3582801
Penicillin-streptomycin-amphotericin B suspension	Sigma-Aldrich	#A5955
Glucose	Sigma-Aldrich	#16301
DMEM, high glucose, with L-glutamine	Gibco	#41965039
Recombinant human BMP-4	Gibco	#120-05ET
Human SCF	Miltenyi Biotec	#130-096-692
Recombinant human VEGF-165	Gibco	#100-20
X-VIVO-15	Fisher Scientific	#16566090
Human M-CSF	Miltenyi Biotec	#130-096-491
Human IL-3	Miltenyi Biotec	#130-093-909
Recombinant human IL-34	Gibco	#200-34
Trypan blue solution 0.4%	Gibco	#15250061
PBS	Life Technologies	#14190169
16% paraformaldehyde	Fisher Scientific	#50-980-487
Sodium azide	Sigma-Aldrich	#S8032
Sucrose	Merck Chemicals	#1076871000
Isopentane	Carl Roth	#3927.2
Tissue-Tek O.C.T. compound	Sakura	#4583
FluorSave reagent Calbiochem	VWR	#345789-20
Normal donkey serum	Jackson ImmunoResearch	#017-000-121
Bovine serum albumin	Sigma-Aldrich	#A4503
Triton X-100	Merck	#T8787
DAPI	Sigma-Aldrich	#D9564
Tween 20	Sigma-Aldrich	#822184
Sodium dodecyl sulfate	Sigma-Aldrich	#8220501000
Glycerol	Supelco	#1040572511
Tris(2-carboxyethyl)phosphine hydrochloride	Sigma-Aldrich	#C4706
D-(−)-fructose	Sigma-Aldrich	#F0127
Urea	Supelco	#1084870500
NaCl	VWR	#1064041000
KCl	VWR	#26764298
NaHCO_3_	Supelco	#1063290500
CaCl_2_	Sigma-Aldrich	#C5670
MgCl_2_	Merck	#1058331000
HEPES	Sigma-Aldrich	#H3375
Glucose	Sigma-Aldrich	#16301
Potassium-D-gluconate	Sigma-Aldrich	#P1847
Phosphocreatine	Sigma-Aldrich	#27920
Na_2_-ATP	Sigma-Aldrich	#A7699-5G
Na_3_-GTP	Sigma-Aldrich	#G8877-100MG
EGTA	Sigma-Aldrich	#E4378
Biocytin	Sigma-Aldrich	#B4261

**Experimental models: Cell lines**		

Human iPSC 929C4	UMC Utrecht	N/A
Human iPSC NL2.1	Harvard Medical School	N/A
Human iPSC Kolf2.c1/Kolf2-C1 ARID2 WT/WT	Sanger Institute	N/A

**Software and algorithms**		

ZEN 2.3 SP1	Zeiss	N/A
Fiji	Schindelin et al.^16^	https://imagej.net/software/fiji/downloads
PatchMaster v.2x92	HEKA Elektronik GmbH	https://scicrunch.org/resolver/RRID:SCR_000034?q=&i=rrid:scr_000034
Clampfit 11.3	Molecular Devices	https://scicrunch.org/resolver/SCR_011323
Python 3.12.7	Python	https://www.python.org/
Prism 9.1.1	GraphPad Software, LLC	https://www.graphpad.com/
Code for converting .dat files in Python	GitHub repository	https://github.com/junzhanj/HEKADatConverter

**Other**		

96-well plate, ultra-low attachment, round bottom	Corning	#7007
6-well hanging cell culture inserts	Millipore	#PTRP06H48
PTFE membrane, 0.45 μm pore size	Millipore	#JHWP04700
6-well plate	Corning	#3516
60 mm petri dish	Greiner Bio-One	#628160
35 mm petri dishes	Greiner Bio-One BV	#627160
P1000 wide bore pipette tips	Sigma-Aldrich	#AXYTF1005WBRS
37 μm reversible strainer	STEMCELL Technologies	#27215
Embedding mold	Simport Scientific	#M475-2
SuperFrost Plus adhesion slides	VWR	#631-0108
Cover glasses (24 × 60 mm)	VWR	#631-1575
iSpacer	SUNJin Lab	#IS213
Borosilicate glass electrode	Harvard Apparatus	#GC150-10
Vibratome blades	Campden Instruments	#752-1-SS

## Materials and equipment

### Materials

**CRITICAL:** Vacuum filter (0.22 μm pore size) all media in the culture hood and keep sterile afterward.

**Table:** 500 mL hES0 medium

Reagent	Final concentration	Volume or Mass
DMEM-F12	–	375 mL
KOSR	20%	100 mL
Fetal Bovine Serum	3%	15 mL
L-Glutamine	2 mM	5 mL
MEM-NEAA	1X	5 mL
2-mercaptoethanol	0.0385 mM	3.5 μL

***Note:*** hES0 medium can be stored at −20°C for up to 6 months, or at 4°C for up to 2 weeks.

**Table:** 500 mL neural medium

Reagent	Final concentration	Volume or Mass
Neurobasal	–	480 mL
B27 without vitamin A (50X)	1X	10 mL
L-Glutamine	2 mM	5 mL
Pen/Strep	100 U/mL	5 mL

***Note:*** Neural medium can be stored at −20°C for up to 6 months, or at 4°C for up to 2 weeks.

**Table:** 50 mL SF-EB medium

Reagent	Final concentration	Volume or Mass
Stemflex	–	50 mL
Y-27632 dihydrochloride	50 μM	500 μL
BMP-4	50 ng/mL	250 μL
SCF	20 ng/mL	10 μL
VEGF	50 ng/mL	25 μL

***Note:*** SF-EB medium can be stored at −20°C for up to 6 months, or at 4°C for up to 2 weeks.

**Table:** 500 mL MacPre medium

Reagent	Final concentration	Volume or Mass
X-VIVO15	–	500 mL
L-Glutamine	2 mM	5 mL
Pen/Strep	100 U/mL	5 mL
2-mercaptoethanol	0.055 mM	5 μL

***Note:*** MacPre medium can be stored at −20°C for up to 6 months, or at 4°C for up to 2 weeks.

**CRITICAL:** Keep MacPre medium in the dark as much as possible, as X-VIVO15 is light sensitive.

**Table:** 500 mL recovery medium

Reagent	Final concentration	Volume or Mass
High glucose DMEM with L-Glutamine	–	445 mL
Fetal Bovine Serum	10%	50 mL
Glucose solution 50% (wt/vol) in Neurobasal	1% (final glucose concentration is 0.5%)	5 mL

***Note:*** Recovery medium can be stored at −20°C for up to 6 months, or at 4°C for up to 2 weeks.

**Table:** 500 mL ALI-CO medium

Reagent	Final concentration	Volume or Mass
Neurobasal	–	480 mL
B27 with vitamin A (50X)	1X	10 mL
Glucose solution 50% (wt/vol) in Neurobasal	1% (final glucose concentration is 0.5%)	5 mL
L-Glutamine	2 mM	5 mL

***Note:*** ALI-CO medium can be stored at −20°C for up to 6 months, or at 4°C for up to 2 weeks.

**Table:** 20 mL blocking solution

Reagent	Final concentration	Volume or Mass
Normal Donkey Serum	10%	2 mL
Bovine Serum Albumin	3%	600 mg
1% Triton X-100 in 1X PBS	–	Fill up to 20 mL

***Note:*** WB1 can be stored at −20°C for up to 6 months, or at 4°C for up to 2 weeks.

**Table:** 1 L WB1

Reagent	Final concentration	Volume or Mass
Tween-20	0.2%	2 mL
10% SDS	0.2%	2 mL
Triton X-100	0.2%	2 mL
Bovine Serum Albumin	0.2%	2 g
1X PBS	–	Fill up to 1 L

***Note:*** Blocking solution can be stored at −20°C for up to 6 months, or at 4°C for up to 2 weeks.

**Table:** 1 L WB2

Reagent	Final concentration	Volume or Mass
10% SDS	0.2%	2 mL
Triton X-100	0.1%	1 mL
Bovine Serum Albumin	0.2%	2 g
1X PBS	–	Fill up to 1 L

***Note:*** WB2 can be stored at −20°C for up to 6 months, or at 4°C for up to 2 weeks.

**Table:** 500 mL 1 M Tris buffer (pH 8)

Reagent	Final concentration	Volume or Mass
Tris	1 M	60.55 g
HCl (stock 36-38% v/v)	3–3.2% (v/v)	42 mL
Distilled water	–	Fill up to 500 mL

***Note:*** Tris buffer can be stored at 18 to 20 °C for up to 6 months.

**Table:** 220 mL FUnGI

Reagent	Final concentration	Volume or Mass
Glycerol	50% (v/v)	110 mL
Distilled water	9.4% (v/v)	20 mL
1 M Tris Buffer, pH 8	10.6 mM	2.2 mL
0.5 M EDTA	1.1 mM	440 μL
Fructose (step 1)	N/A	50 g
Fructose (step 2)	2.5 mM (after step 2)	49 g
Urea	2.5 mM	33.1 g

***Note:*** FUnGI solution can be stored at 4 °C in the dark for up to 4 weeks.

**CRITICAL:** Add fructose in two steps to allow for proper dissolvement.

**Table:** Artificial cerebrospinal fluid (aCSF; 300-310 mOsm, pH 7.35)

Reagent	Final concentration
NaCl	124 mM
KCl	2.5 mM
NaH_2_PO_4_	1 mM
NaHCO_3_	26 mM
CaCl_2_	2.5 mM
MgCl_2_	1.3 mM
HEPES	5 mM
Glucose	11 mM

***Note:*** aCSF can be prepared freshly or stored at 4 °C for up to 1 week.

**Table:** Internal solution (300-310 mOsm, pH 7.35, liquid junction potential −12 mV)

Reagent	Final concentration
K-Gluconate	139 mM
KCl	5 mM
HEPES	10 mM
Phosphocreatine	10 mM
MgCl2	2 mM
Na_2_-ATP	4 mM
Na_3_-GTP	0.3 mM
EGTA	0.2 mM

***Note:*** Internal solution can be stored at −20°C for up to 3 months and kept on ice during the day of the recording.

**CRITICAL:** Filter the final internal solution three times using a 0.22 μm pore size filter to minimize debris and prevent internal blockage of the patch electrode.

**Table:** Equipment

Equipment type	Parts	Specifications
Vibratome		VT1000S (Leica)
Cryostat		CM1950 (Leica Biosystems)
Confocal microscope		LSM 880, AxioObserver
	20x air objective	Plan-Apochromat 20x/0.8 (Olympus)
	63x oil objective	Plan-Apochromat 63x/1.40 oil DIC M27 (Olympus)
Upright Microscope (electrophysiology)		SliceScope Pro 6000 (Scientifica)
	4x air objective	RMS4x- Plan Achromat, 0.10 NA (Olympus)
	40x water immersion objective	LUMPLFLN 40XW (Olympus)
Electrophysiology set-up	Amplifier	EPC 10 USB Double (HEKA Elektronik)
	Micromanipulator	PatchStar (Scientifica)
	Control Cube	ACCi UI (Scientifica)
	Headstage	EPC 10 USB probe 2 (HEKA Elektronik)
	Heater Controller	TC-324 (Warner Instrument Corporation)
	In-Line Solution Heater	SH-27B (Warner Instrument Corporation)
	Basic Peristaltic Pump	BT100-3J (Longer)

## Step-by-step method details

**Figure 2:**
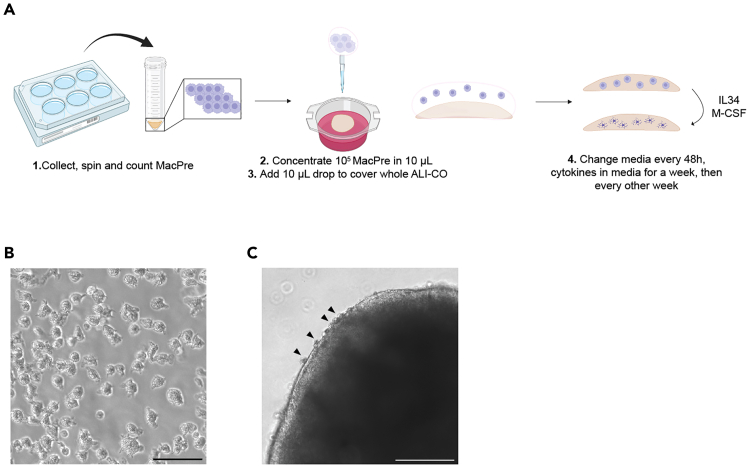
MacPre integration into ALI-COs (A) Schematic showing the different steps of MacPre integration protocol. Created with BioRender.com. (B) Bright-field image of 4-weeks-old MacPre floating in medium before collection. Scale bar, 100 μm. (C) Bright-field image showing MacPre (black arrowheads) at the edges of a MG-ALI-CO two days after integration. Scale bar, 250 μm.

**Figure 3:**
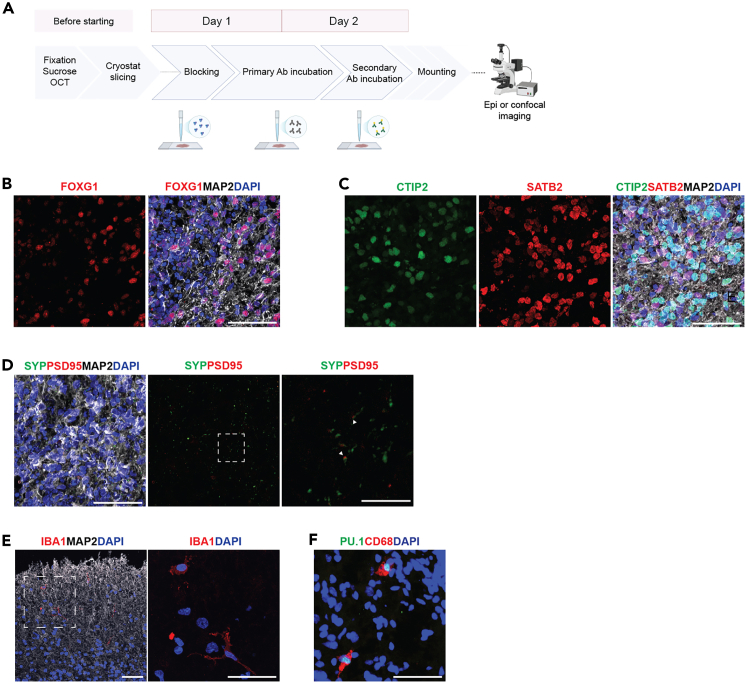
2D immunohistochemistry on MG-ALI-CO sections (A) Schematic overview of the different steps of 2D IHC. Created with BioRender.com. (B) DIV140 ALI-CO immunostained for forebrain progenitors (FOXG1) and a dendritic marker (MAP2). DAPI stains nuclei. Scale bar, 50 μm. (C) DIV140 ALI-CO immunostained for deep-layer cortical neurons (CTIP2), upper-layer cortical neurons (SATB2), and MAP2. DAPI stains nuclei. Scale bar, 50 μm. (D) DIV140 ALI-CO immunostained for a pre-synaptic marker (SYP), a post-synaptic marker (PSD-95) and MAP2. DAPI stains nuclei. Boxed area is shown at higher magnification on the right and shows co-localization of SYP and PSD-95 (white arrowheads). Scale bar, 50 μm/10 μm. (E) DIV140 MG-ALI-CO (6 weeks after MacPre integration) immunostained for microglia (IBA1) and MAP2. DAPI stains nuclei. Boxed region is shown at higher magnification in the right panel. Scale bar, 50 μm. (F) DIV140 MG-ALI-CO (6 weeks after MacPre integration) immunostained for microglia (PU.1, CD68). DAPI stains nuclei. Scale bar, 50 μm.

**Figure 4:**
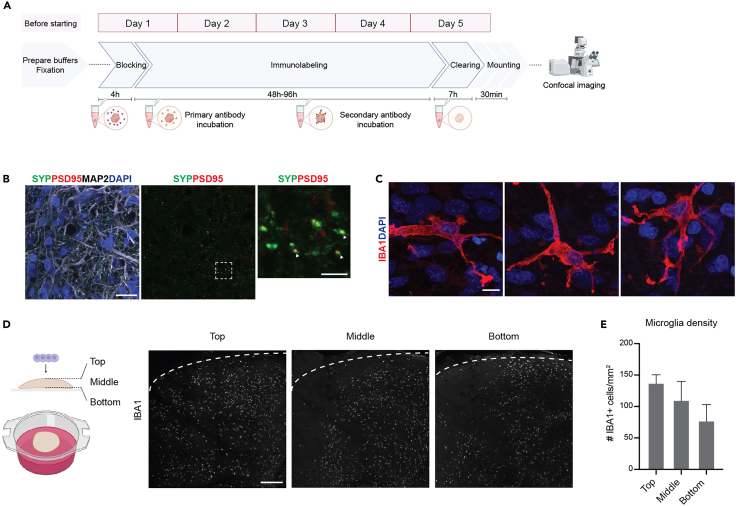
3D immunohistochemistry and tissue clearing of MG-ALI-COs (A) Schematic overview of the different steps of 3D IHC followed by tissue clearing. Created with BioRender.com. (B) DIV140 ALI-CO immunostained for SYP, PSD-95 and MAP2. DAPI stains nuclei. Boxed region is shown at higher magnification in the right panel and shows co-localization of SYP and PSD-95 (white arrowheads). Scale bars, 25 μm/5 μm. (C) Individual microglial cells from a DIV150 MG-ALI-CO (8 weeks after MacPre integration; 2∗10^5^ integrated MacPre) immunostained for IBA1. DAPI stains nuclei. Scale bar, 10 μm. (D) Schematic showing microglia distribution analysis in DIV150 MG-ALI-CO (8 weeks after MacPre integration; 2∗10^5^ integrated MacPre) with representative images of the top, middle, and bottom 20 μm thick sections to the right. Scale bar, 500 μm. (E) Microglia density quantification in 15 μm thick areas from top, middle, and bottom sections of DIV150 MG-ALI-COs (8 weeks after MacPre integration; 2∗10^5^ integrated MacPre). Data are represented as means + SEM.

**Figure 5:**
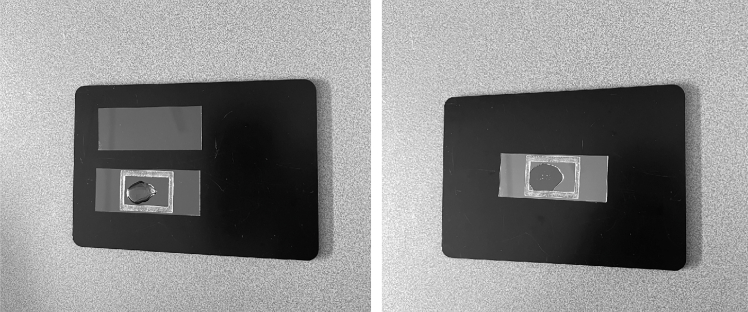
Mounted MG-ALI-CO in FUnGI solution Thin cover glasses with iSpacer and a drop of 100% FUnGI. A MG-ALI-CO is placed in the middle of the FUnGI drop (left). Cover glass mounted on the iSpacer and FUnGI drop containing MG-ALI-CO (right).

**Figure 6:**
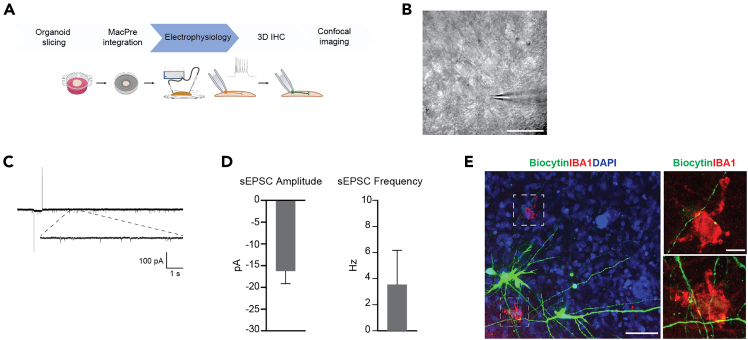
Electrophysiological recording of MG-ALI-CO (A) Schematic overview of the different steps preceding and following electrophysiological recording. Created with BioRender.com. (B) MG-ALI-CO bright-field image as seen from the electrophysiology setup microscope. Scale bar, 50 μm. (C) Representative trace of an individual neuron’s electrophysiological recording. (D) Quantification of sEPSC amplitude and frequency from individual neurons in DIV120-170 MG-ALI-CO (6 weeks after MacPre integration; 10^5^ integrated MacPre). Data are represented as means + SEM, N=12 cells (1 cell = 1 MG-ALI-CO). (E) MG-ALI-CO immunostained for patched neurons (biocytin) and IBA1. DAPI stains nuclei. Boxed areas are shown at higher magnification in the two right panels and show the close proximity of biocytin-filled neurons and microglia. Scale bars, 50 μm/ 10 μm.

**Figure 7:**
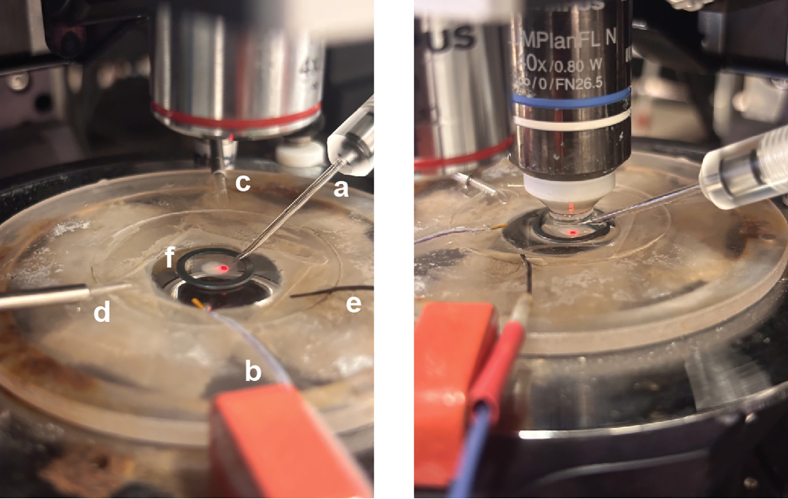
MG-ALI-CO in the electrophysiology setup MG-ALI-CO placed in the recording chamber of the electrophysiology set-up. Left image shows MG-ALI-CO placed under a 4x objective to screen the sample and locate an area of interest for patching, together with patch pipette (a), temperature probe (b), CSF inlet (c) and outlet (left) (d), reference electrode (e) and harp slice grid (f). Right image shows a ready-to-patch MG-ALI-CO placed under a 40x objective.

**Figure 8:**
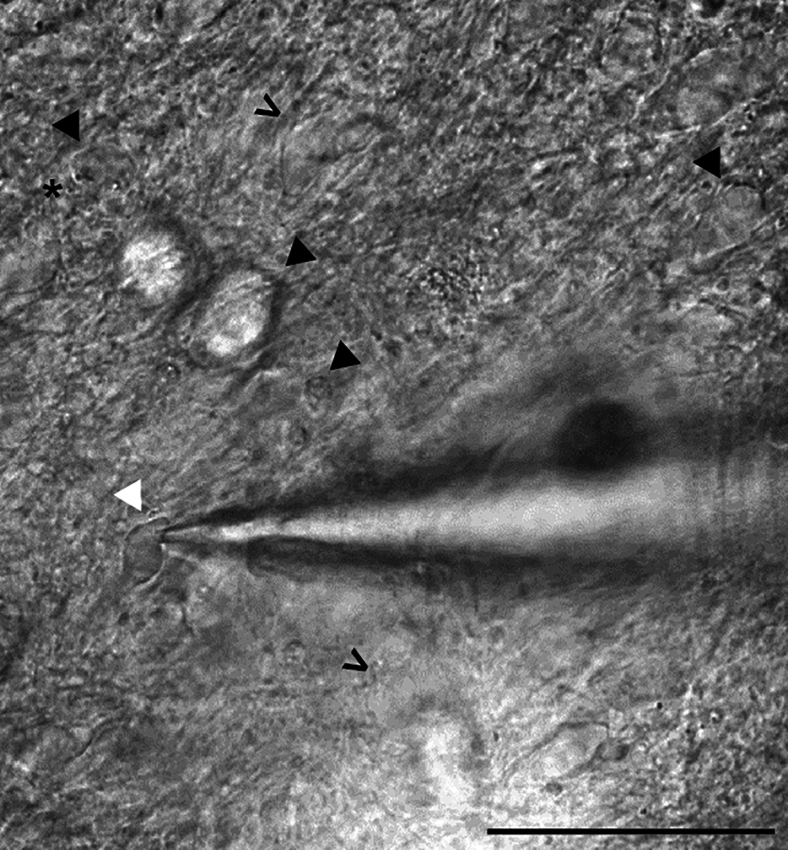
Bright-field image of a MG-ALI-CO containing multiple candidate cells for patching Patch pipette sealed to a neuron (white arrowhead) surrounded by good candidate cells (open arrowheads) and poor candidate cells (black arrowheads) for patching. Poor quality cells contain structures of putative fragmented DNA (star), dramatically enlarged soma sizes, or soma sizes <10 μm, indicating unhealthy or non-neuronal cells. Scale bar, 50 μm.

This section describes the protocol to integrate MacPre in ALI-COs (Figure 2), followed by protocols for three different experimental analyses: 2D IHC (Figure 3), 3D IHC (Figures 4 and 5), electrophysiological recordings (Figures 6, 7, and 8), and two confocal imaging examples.

***Note:*** The experimental analysis can be performed in parallel, and are not proposed as subsequent steps. They are designed to be independent experiments.

### Macrophage precursor integration into ALI-COs

**Timing: 2 h**

The following section describes the steps needed to integrate MacPre into ALI-COs. It takes one full 6 well-plate, with ∼15 YS-EBs per well, where all collected MacPre from one plate are pooled together, and DIV90-100 ALI-COs as starting material. If collection is performed between week 2–3 to 5, a sufficient number of cells for integration into at least 10 ALI-COs will be present in the medium 5–6 days after the last media change.

***Note:*** MacPre are obtained as described in the “before you begin” section. MacPre typically arise in the medium between 2 to 3 weeks after YS-EBs plating (depending on the iPSC line and batch). If medium is changed weekly, YS-EBs produce MacPre cells up to week 8. However, restricting collection up to week 5 is highly advisable, as MacPre cells visibly change morphology (*i.e.* swelling) as culture time increases. Likewise, published protocols that use MacPre for xenotransplantation into the mouse brain report reduced grafting efficiency when older MacPre are used.^17^

**CRITICAL:** Before starting collection, cultures should be examined under the microscope to confirm the presence of MacPre in the media (Figure 2B).

1. Place ALI-COs that will be used for integrations on a separate hanging cell culture insert in ALI-CO medium supplemented with 10 ng/mL M-CSF and 100 ng/mL IL-34.

**CRITICAL:** Only use ALI-COs ≥ 4 mm in diameter for MacPre integration as smaller ALI-COs will not survive the integration. Small ALI-COs will start dying 2–3 weeks after integration.

2. Place a 37 μm cell strainer in a 50 mL Falcon tube and add 1 mL of MacPre medium through the strainer.
3. Transfer 80% of MacPre culture medium from each well through the cell strainer using a P1000 pipette.

***Note:*** Avoid taking large pieces of tissue or YS-EBs when transferring medium. If this cannot be avoided, reversible strainers can be used to transfer the YS-EBs back into the wells once collection is finished.

***Note:*** In wells with a large number of MacPre, a ‘cloud’ of cells can be observed. Collect as much of this cloud as possible.

4. Rinse the strainer with 1 mL MacPre medium and collect any remaining liquid from underneath the strainer.
5. Centrifuge cells for 5 minutes at 200 rcf at 18 to 20°C.
6. Resuspend pellet in 200 μL of ALI-CO medium supplemented with 10 ng/mL M-CSF and 100 ng/mL IL-34 and perform live cell counting using Trypan Blue.

***Note:*** If the pellet is very large and cell counting is difficult due to high cell density, dilute cells with additional medium.

***Note:*** One 6-well plate typically yields between 1–10 million MacPre, depending on the iPSC line.

**CRITICAL:** MacPre viability should be above 75% to ensure optimal differentiation and to avoid compromising ALI-CO quality due to large numbers of dead cells.

7. Based on the calculated cell density, adjust the concentration to 10^4^ cells/μL.
8. Place a 10 μL drop (10^5^ cells) on top of the ALI-CO and wait for approximately 2 minutes before returning the culture plates to the incubator.

***Note:*** A range of 10^5^ to 2∗10^5^ MacPre per ALI-CO can be integrated depending on MacPre availability.

***Note:*** Drop volume can be 10 ± 5 μL depending on ALI-CO size. The MacPre-containing drop should cover the entire ALI-CO but not overflow. If cell density is too low to reach 10^4^ cells/μL, two or three sequential drops can be added until a total amount of 10^5^- 2∗10^5^ cells are seeded on top of the ALI-CO. For this, 3 to 5-minute intervals between each drop are recommended, or until drop has visible sunken into the ALI-CO.

9. After 48 h, change ALI-CO medium completely, supplemented with fresh 10 ng/mL M-CSF and 100 ng/mL IL-34, every other day for one week.
10. Continue changing all medium every other day and supplement with fresh M-CSF and IL-34 every other week during one medium change.

***Note:*** MG-ALI-CO medium change can be performed as for ALI-CO, since MacPre rapidly attach to the tissue after the integration procedure.

***Note:*** Here, MG-ALI-COs are assessed only up to two months after MacPre integration (DIV140-150 MG-ALI-COs). Researchers aiming to culture MG-ALI-COs for a longer period of time should assess whether microglia density, morphology and marker expression are maintained.

### 2D immunohistochemistry of MG-ALI-COs

**Timing: 4 days**

This section provides a description of the steps required to perform a 2D IHC on MG-ALI-COs (Figure 3A).

***Note:*** Cryosectioning of MG-ALI-COs followed by 2D IHC offers the possibility to rapidly and efficiently stain for multiple cell types or cell structures within one MG-ALI-CO.

11. Fix and dehydrate the MG-ALI-CO.
  a. Fix the organoids.
    i. Collect MG-ALI-COs by carefully lifting one side of the membrane with sterile tweezers and put the MG-ALI-CO in a labeled 2 mL Eppendorf tube with 1 mL 1X PBS (one MG-ALI-CO per Eppendorf tube).
      ***Note:*** Keep MG-ALI-COs attached to the membrane during fixation to prevent significant shape changes.
    ii. Replace 1X PBS with 1 mL 4% PFA solution in PBS.
    iii. Incubate MG-ALI-COs in PFA for 12 to 16 hours at 4°C on a shaker.
    iv. The following day, remove the PFA and wash one time briefly and two times for 5 minutes with 1X PBS.
      **Pause point:** Fixed MG-ALI-COs can be stored long-term at 4°C in a 2 mL Eppendorf tube with 1X PBS. When storing samples for >1 month, add NaN_3_ (0.05%) to the PBS to prevent bacterial and fungal infections.
  b. Transfer the MG-ALI-COs to a 6 cm petri dish filled with 1X PBS.
  c. Carefully remove MG-ALI-COs from their membrane by horizontally dispensing PBS to the sides of the tissue using a P1000 pipette.
    **CRITICAL:** When removing MG-ALI-COs from the membrane, do not rupture the tissue. The MG-ALI-COs should detach easily. If not, try to put the P1000 tip as close as possible to the MG-ALI-CO while pressing down on the membrane with the tip, preventing the membrane from moving. The degree of easy detachment of MG-ALI-COs is dependent on the iPSC line used.
  d. Transfer the MG-ALI-COs into a 15 mL Falcon using a P1000 pipette with a wide bore (or cut) tip and add 5 mL 30% sucrose in 1X PBS for 12 to 16 hours at 4°C or until the MG-ALI-COs have sunken to the bottom of the tube.
12. Embed the organoids.
  a. Prepare a glass beaker with isopentane embedded in a bucket with dry ice.
  b. Ensure that the temperature of the isopentane is between −30°C and −50°C.
  c. Collect the MG-ALI-CO using a P1000 pipette with a wide bore (or cut) tip and place it in an embedding mold.
  d. Remove excess sucrose from the mold using a P200 pipette without touching the MG-ALI-CO.
  e. Fill the mold with Tissue-Tek O.C.T. Compound. Prevent the formation of air bubbles in the O.C.T. Compound.
  f. Gently move the MG-ALI-CO to the middle of the mold using a P10 tip.
    ***Note:*** When adding more than one MG-ALI-CO to the same mold, ensure that the MG-ALI-COs are positioned next to each other in the same horizontal plane.
  g. Carefully place the embedding mold in the cold isopentane until the O.C.T. Compound is completely frozen (1–2 minutes).
  h. Keep the embedded MG-ALI-COs on dry ice before storage at −80°C.
13. Cryosection the embedded organoids.
  a. Set the cryostat chamber temperature to −19°C, and the object temperature to −17°C.
  b. Mount the block of Tissue-Tek O.C.T. Compound with the MG-ALI-CO on the holder using a droplet of O.C.T. Compound.
  c. Let the embedded block adjust to the chamber temperature for approximately 30 minutes.
  d. Slice the MG-ALI-CO into 20 μm thick sections. Mount sections on SuperFrost Plus adhesion slides.
  e. Let sections on the slides air-dry for at least 1 hour before storage at −80°C.

***Note:*** If sections on the slides dry slowly, blow-dry the sections before storage.

**Pause point:** Slides can be stored long-term at −80°C before proceeding to the IHC procedure.

14. Perform IHC on the MG-ALI-CO sections.
  a. Block tissue with Blocking Solution.
    i. Collect the slides from the −80°C and let them thaw at 18 to 20°C for at least 15 minutes (up to 2 hours).
    ii. Put the slides in a container filled with 1X PBS on a shaker for 10 minutes.
    iii. Incubate the slides in an incubation box for approximately 1 hour with Blocking Solution.
  b. Incubate the slides with the primary antibody solution.
    i. Prepare a humidified, covered incubation box in which the slides can be on a flat surface.
      ***Note:*** If primary antibodies are conjugated to a fluorophore, keep the slides in the dark as much as possible from this step onwards.
    ii. Discard the Blocking Solution and cover the slides with 300 μL per slide of primary antibody diluted in Blocking Solution.
    iii. Incubate for 12 to 16 hours at 4°C.
  c. Incubate the slides with the secondary antibody solution in the incubation box.
    i. Wash slides three times with 1X PBS, one brief wash and two times for 5 minutes.
      ***Note:*** If secondary antibodies are conjugated to a fluorophore, keep the slides in the dark as much as possible from this step onwards.
    ii. Incubate slides with 300 μL per slide of secondary antibodies diluted in Blocking Solution for 1 hour at 18 to 20°C.
    iii. Rinse slides with 1X PBS.
    iv. Incubate with 300 μL DAPI (1 μg/mL in 1X PBS) per slide for 15 minutes at 18 to 20°C.
    v. Wash slides two times for 5 minutes with 1X PBS.
  d. Remove PBS from the slide and mount using FluorSave reagent and a cover glass.

### 3D immunohistochemistry and tissue clearing of MG-ALI-COs

**Timing: 5 days**

This section provides a detailed description of the steps needed to perform 3D IHC of whole MG-ALI-COs, followed by tissue clearing (Figure 4A). This protocol is adapted from Van Ineveld et al., 2022,^18^ which utilizes fructose, urea and glycerol clearing solution for imaging (FUnGI).

***Note:*** 3D IHC provides the possibility to obtain a complete image of cell-cell interactions and connections in the MG-ALI-CO, which is limited in 2D IHC due to tissue sectioning.

15. Perform fixation as described in step 1 and 2 of the “2D immunohistochemistry of MG- ALI-COs” protocol.

***Note:*** It is advised to prepare WB1, WB2 and FUnGI before starting the blocking step, since preparation time of these buffers takes a full day.

16. Block tissue with WB1.
  a. After removing MG-ALI-COs from the membrane, transfer them to an Eppendorf tube and remove all PBS.
  b. Add 500 μL of WB1 to the tube and gently invert to ensure that the MG-ALI-CO is submerged in the WB1.
  c. Take a 50 mL tube and put small pieces of tissue paper at the bottom.
  d. Gently invert the Eppendorf tube and place it upside down on the tissue paper in the 50 mL tube.
  e. Add tissue paper pieces around and on top of the Eppendorf to ensure it cannot move.
    ***Note:*** If multiple MG-ALI-COs are being processed, add another Eppendorf tube to the 50 mL tube and repeat until the tube is full. Stabilize Eppendorf tubes with tissue paper pieces and close the 50 mL tube.
  f. Place the 50 mL tube on a shaker or roller bank at 18 to 20°C for 4 hours.
    **Pause point:** After blocking, MG-ALI-COs can be stored in WB2 for 2 days at 4°C. However, the protocol should be continued as soon as possible.
    ***Note:*** When removing the Eppendorf tube from the 50 mL tube, remove most of the tissue paper pieces first. This prevents the lid from the Eppendorf tube from accidentally opening.
17. Perform IHC on the MG-ALI-COs.
  a. Prepare primary antibody mix in WB2. For each MG-ALI-CO, 500 μL of antibody mix is required.
    **CRITICAL:** When preparing primary antibody mix, only add half of the desired amount of antibody. There is a replenishing step the following day during which the second half is added. For example, for an antibody that has a working dilution of 1:1000 and needs *e.g.,* 5 μl of undiluted antibody, add 2.5 μl on the first day (1:2000 dilution) and 2.5 μl on the next day (final dilution of 1:1000).
    ***Note:*** When working with conjugated antibodies, add these in the secondary antibody mix.
    i. Remove WB1 from the Eppendorf tube without disrupting the MG-ALI-CO and replace with primary antibody mix.
    ii. Invert the Eppendorf tube and place back in the 50 mL tube.
    iii. Place the 50 mL tube on a shaker or roller bank and incubate for 12 to 16 hours at 18 to 20°C .
  b. The following day, add the other half of the total amount of primary antibodies directly to the Eppendorf tube with the MG-ALI-CO to reach the final antibody concentration.
  c. Return the 50 mL tube back to the shaker or roller bank and incubate for 12 to 16 hours at 18 to 20°C.
  d. Wash MG-ALI-CO and prepare secondary antibody mix.
    i. Take the Eppendorf tube from the 50 mL tube and gently remove the primary antibody mix.
    ii. For washing, add 500 μL WB2 and put Eppendorf tube back in the 50 mL tube.
    iii. Return the 50 mL tube back to the shaker or roller bank for 1 hour at 18 to 20°C.
    iv. Repeat this step 2 times (*i.e.,* in total of 3 washes of at least 1 hour each).
      ***Note:*** Ensure that all liquid is removed between washing steps to prevent non-specific primary antibody binding and avoid tissue damage during the process.
      **Pause point:** After washing, MG-ALI-COs can be stored in WB2 for 2 days at 4°C. However, the protocol should be continued as soon as possible.
    v. Prepare secondary antibody mix in WB2. For each MG-ALI-CO, 500 μL antibody mix is required, including 1 μg/mL DAPI.
      **CRITICAL:** When preparing secondary antibody mix, only add half of the desired amount of antibody. Similarly as with the primary antibodies, there is a replenishing step the following day during which the second half is added.
    vi. Remove WB2 from the Eppendorf tube, without disrupting the MG-ALI-CO, and replace with secondary antibody mix.
      ***Note:*** Keep MG-ALI-COs in the dark as much as possible from this step onwards.
    vii. Invert Eppendorf tube and return to the 50 mL tube.
    viii. Wrap the 50 mL tube in aluminum foil to keep the MG-ALI-COs in the dark.
    ix. Put the 50 mL tube on the shaker or roller bank and incubate for 12 to 16 hours at 18 to 20°C.
  e. Add the other half of the total amount of secondary antibodies, including DAPI, to the Eppendorf tube with the MG-ALI-CO to reach the final antibody concentration.
  f. Return the 50 mL tube, wrapped in aluminum foil, to the shaker or roller bank.
  g. Incubate for 12 to 16 hours at 18 to 20°C.
18. Clear the tissue.
  a. Wash the MG-ALI-COs.
    i. Gently remove the secondary antibody mix.
    ii. For washing, add 500 μL WB2 and return the Eppendorf tube to the 50 mL tube.
    iii. Put the 50 mL tube on the shaker or roller bank for 1 hour at 18 to 20°C.
    iv. Repeat this step 2 times (*i.e.,* in total of 3 washes of at least 1 hour each).
  b. Perform FUnGI procedure.
    i. Prepare 33% and 66% FUnGI mixes in WB2 (from the 100% FUnGI stock solution).
    ii. Homogenize the mixes by vortexing briefly.
    iii. Keep the required amount of 33% and 66% mixes and 100% FUnGI solution at 18 to 20°C.
      ***Note:*** Bring the 100% FUnGI solution to 18 to 20°C before mixing to reduce viscosity. Dispense FUnGI solution slowly to avoid bubbles. Use cut P1000 tips if necessary.
    iv. Remove WB2 from the Eppendorf tube, without disrupting the MG-ALI-CO, and replace with 500 μL 33% FUnGI mix.
    v. Return the Eppendorf tube to the 50 mL tube.
    vi. Put the 50 mL tube, wrapped in aluminum foil, on the shaker or roller bank and incubate for 1 hour at 18 to 20°C.
      ***Note:*** Always freshly prepare the 33% and 66% FUnGI mixes on the day of clearing, as BSA in WB2 should not be kept at 18 to 20°C for >24 hours.
      ***Note:*** Throughout the clearing process MG-ALI-COs will gradually become translucent. Take extra care when removing liquid from this step onward to avoid accidentally damaging translucent parts of the MG-ALI-COs.
    vii. Carefully remove 33% FUnGI mix and replace with 500 μL of 66% FUnGI mix.
    viii. Return the Eppendorf tube to the 50 mL tube.
    ix. Put the aluminum-wrapped 50 mL tube on the shaker or roller bank for 1 hour at 18 to 20°C.
    x. Carefully remove 66% FUnGI mix and replace with 500 μL of 100% FUnGI.
    xi. Return the Eppendorf tube to the 50 mL tube.
    xii. Put the aluminum-wrapped 50 mL tube to the shaker or roller bank for 3 hours at 18 to 20°C.
      **Pause point:** Stained and cleared MG-ALI-COs can be stored in the dark for ∼1 to 2 weeks at 4°C before proceeding to tissue mounting.
      ***Note:*** The FUnGI clearing process can be reversed as shown in Van Ineveld et al., 2022.^18^
19. Mount cleared MG-ALI-COs between two cover glasses (Figure 5).
  ***Note:*** Use two thin (0.16 mm) cover glasses to mount the MG-ALI-COs.
  a. Place a thin cover glass on a flat and clean surface.
  b. Take a double-sided sticky iSpacer, take off the sticker cover on one side and place the iSpacer in the center of the cover glass.
  c. Gently press on the edges of the iSpacer to ensure it fully attaches to the cover glass.
  d. Gently remove the sticker cover on the top side of the iSpacer using tweezers.
  e. Place a drop of approximately 200 μL 100% FUnGI (RT) on one side of the cover glass within the iSpacer.
    ***Note:*** The droplet of 100% FUnGI should be sufficiently large to fully cover the cleared MG-ALI-CO without filling up the iSpacer, as this can cause leakage (Figure 5).
  f. Carefully place the cleared MG-ALI-CO in the droplet of 100% FUnGI within the iSpacer using a P1000 pipette with a cut tip.
    ***Note:*** The MG-ALI-CO will be completely or almost completely translucent depending on its size. It is therefore difficult to locate in the Eppendorf tube and care should be taken to not damage the MG-ALI-CO during lifting and positioning.
  g. Gently press a second cover glass on the iSpacer, starting at the side where the MG-ALI-CO is located. Slowly lower the glass on the iSpacer, from the side with the MG-ALI-CO to the opposite side. This allows FUnGI to evenly spread over the iSpacer.
  h. Ensure the MG-ALI-CO is fully covered by and submerged in the FUnGI.
  i. Carefully press the top cover glass down right above the iSpacer rim to tightly attach the cover glass.
  j. The mounted MG-ALI-CO can be imaged immediately or stored long-term in the dark at 4°C.
    ***Note:*** Store the mounted MG-ALI-COs in the dark in a horizontal position to prevent leakage of FUnGI from the iSpacer. MG-ALI-COs need to remain submerged in FUnGI.
    ***Note:*** Ensure that the mounted MG-ALI-CO is in contact with the cover glasses at both sides to keep it firmly in place. Otherwise, the tissue might ‘float’ in FUnGI, leading to tissue drift during imaging.
    **CRITICAL:** FUnGI viscosity is affected by temperature changes. When mounted MG-ALI-COs are stored at 4°C, keep them at 18 to 20°C (in the dark) for approximately 30 minutes before imaging.

### Confocal imaging

**Timing: variable**

***Note:*** For identifying cell types or gross cell morphology, imaging of 2D stained MG-ALI-COs with an epi-fluorescent microscope is sufficient. However, visualization of cell-cell interactions or whole-cell morphologies usually require 3D IHC. We recommend the use of confocal microscopy for imaging the 3D stained and cleared MG-ALI-COs to reduce background fluorescence. This section provides an overview of confocal imaging of synapses and microglia. Instructions for imaging of synapses and microglia apply to both 2D and 3D IHC, unless indicated otherwise.

***Note:*** When investigating synapses, we recommend the use of DIV140 or older MG-ALI-COs, as pre- and post-synaptic markers are abundantly present from this time point onwards.

20. Image pre- and post-synaptic compartments (Figures 3D and 4B).
  ***Note:*** These images can be used to quantify, for example, synapse number and size or to assess colocalization of pre- and post-synaptic markers.
  a. Image MG-ALI-COs using a confocal microscope.
    i. Use MAP2, SYP, and PSD-95 antibodies to identify dendrites and pre- and post-synaptic compartments, respectively (Figures 3D and 4B). Use DAPI to label cell nuclei.
    ii. Use a 63X objective and obtain z-stacks of at least one cell thickness, with approximately 0.3 μm intervals between z-slices. Use DAPI staining to navigate through the z-stack.
      ***Note:*** Do not capture too many z-slices or very thick z-stacks, as this will increase background fluorescence.
      ***Note:*** Optimal interval size between z-slices depends on multiple factors (*e.g.* confocal microscope axial resolution). As the interval indicated here is influenced by the confocal microscope used in this protocol, researchers should accurately determine this value based on their imaging set-up.
      **CRITICAL:** High laser power (∼70 to 100%) is required to capture the signal of the conjugated single-domain PSD-95 antibody used in this protocol.
  b. Perform image post-processing using FIJI software.
    i. Adjust the brightness and contrast of the channels with the synaptic markers, so that synaptic puncta are visible with low background signals.
21. Image microglia.
  ***Note:*** Assessment of microglia marker expression can be achieved by using 2D IHC (Figures 3E and 3F), but we recommend 3D IHC for performing microglia cell morphology and distribution analyses (Figures 4C–4E).
  a. Image the MG-ALI-COs with a confocal microscope. Use MAP2 and IBA1 antibodies and DAPI to visualize dendrites, microglia and nuclei, respectively (Figures 3E and 4C–4E).
    ***Note:*** MAP2 is not essential but helps to assess tissue quality. Alternative neuronal markers (*e.g.,* TUBB3) can be used instead of MAP2.
    ***Note:*** PU.1 and CD68 antibodies can be used to identify macrophages and microglia (Figure 3F). The analysis described here requires a marker that labels the entire microglial cell, such as IBA1.
  b. To assess microglia morphology, image individual cells using a 63x objective, generating a z-stack thick enough to encompass the cell of interest.
    i. A z-slice interval of 0.75 μm is sufficient to capture detailed morphologies (Figure 4C).
    ii. Use the IBA1 channel to navigate through the z-slices and ensure that there is a nucleus (DAPI) that can be assigned to that cell.
  c. To assess microglia distribution and density, image the entire MG-ALI-CO using a 10x objective.
    i. Acquire multiple tiles and a z-stack that captures the entire MG-ALI-CO. This can take several hours.
    ii. A distance of 6.8 μm between z-slices is sufficient to visualize microglia distribution and density, as well as for capturing gross cell morphology.
    iii. Imaging of the IBA1 channel is sufficient to assess these parameters.
  d. Post-process and analyze morphology in FIJI software.
    i. Analyze microglia morphology by generating a maximum projection of each image. Alternatively, software like Imaris can be used to visualize the entire cell in 3D.
  e. Post-process and analyze microglia distribution in FIJI software (Figures 4D and 4E).
    i. Divide the z-stacked MG-ALI-CO image into three sections: top, middle and bottom.
    ii. Within each section, select a number of adjacent z-slices of approximately 15–20 μm in total to make a maximum projection. Select representative z-slices of each corresponding section.
    iii. Select 3 ROIs of 500 × 500 μm within the representative maximum projection of each section and count the number of microglia per ROI to obtain cell density (cells per mm^2^) (Figures 4D and 4E). Counting can be performed manually or by using the “analyze particles” plugin.

### Electrophysiological recording of MG-ALI-COs

**Timing: 2 days**

In this section, a comprehensive overview of the steps required to obtain electrophysiological recordings using the patch-clamp technique on MG-ALI-COs is provided, with a focus on spontaneous excitatory postsynaptic currents (sEPSCs; Figure 6).

***Note:*** Performing electrophysiological recordings on neurons allows for detailed understanding of their functional properties.^19^

22. Prepare the electrophysiology experiment setup.
  a. Prepare artificial cerebrospinal fluid (aCSF) and internal solution on the day of recording.
    **CRITICAL:** To prevent nucleotide degradation, keep the internal solution on ice throughout the recording day.
    ***Note:*** Using a single batch of internal solution helps minimize variability between different recording days of a single experiment.
  b. Add 0.5% biocytin to the internal solution to enable visualization and morphological reconstruction of cells after recording.
    **CRITICAL:** Add MgCl_2_ and CaCl_2_ separately from a 1 M stock solution after oxygenation of aCSF with 95% O_2_ and 5% CO_2_ for at least 10 minutes. This is used to prevent precipitation of insoluble carbonate salts due to changes in pH.
    ***Note:*** Keep freshly prepared aCSF at 37°C ± 1°C before starting the recordings.
  c. Configure the settings of the amplifier, apply a low-pass filter at 2.9 kHz with a 4-pole Bessel filter. Digitize the recordings at 20 kHz with an EPC10 dual path-clamp USB amplifier using PatchMaster v.2x92 software.
    ***Note:*** Similar settings can be configured with any chosen amplifier.
  d. Collect the MG-ALI-CO and prepare it for patching.
    i. Prepare a 3.5 cm cell culture dish with fresh aCSF at 37°C ± 1°C, oxygenated with 95% O_2_ and 5% CO_2_.
    ii. Collect the MG-ALI-CO by carefully lifting a corner of the membrane with sterile tweezers and place the MG-ALI-CO, still attached to the membrane, in the dish with aCSF.
      ***Note:*** Avoid touching the aCSF with the tweezer.
    iii. The MG-ALI-CO can now be transferred to the electrophysiology set-up without taking measures to ensure sterility. The MG-ALI-CO will be fixed directly after patching.
    iv. Place the MG-ALI-CO, still attached to the membrane, in the recording chamber of the electrophysiology set-up (Figure 7).
    v. Continuously oxygenate aCSF at 37°C ± 1°C at a flow rate of 2.5 mL/min using a peristaltic pump.
      ***Note:*** The electrophysiology set-up should also include an upright microscope with oblique illumination and a 40x water immersion objective to visualize cells.
23. Start the patch-clamp recording.
  a. Identify candidate cells for patching located within ± 500 μm from the edge of the MG-ALI-CO (Figure 8).
    ***Note:*** Excitatory corticofugal projection neurons develop in cortical organoids, located mostly within 500 μm of the edge from 3 months onward.^20^ Even though Uzquiano and colleagues investigated whole organoids rather than MG-ALI-COs, these observations are taken into account. Therefore, only neurons lying ± 500 μm from the edge of the MG-ALI-CO are considered for patching.
    i. Use Borosilicate glass electrodes with a resistance of 3 to 5 MΩ for patching.
    ii. Compensate pipette resistance before patching.
    iii. Patch cells in voltage-clamp configuration at −65 mV resting membrane potential. Optionally, compensate for the liquid junction potential at this step.
    iv. Immediately after cell break in, switch to current-clamp (I = 0 pA) configuration to assess resting membrane potential (V_m_).
      **CRITICAL:** Discontinue if cell V_m_ is > −40 mV.
    v. Switch back to voltage clamp at −65 mV, let the cell rest for 2 minutes to ensure stability during recording.
      **CRITICAL:** Discontinue if leak current (I_leak_) < − 150 pA or series resistance (R_s_) > 25 MΩ.
  b. Measure sEPSCs to assess synaptic connectivity.
    i. Run an episodic voltage clamp protocol for 5 minutes at −65 mV resting membrane potential.
    ii. Start a new sweep every 10 seconds, reaching 30 x 10 seconds in total.
    iii. In each sweep, perform a 500 ms −5 mV step at t = 1 s to allow for offline analysis of passive membrane properties (*e.g.*, series resistance (R_s_), input resistance (R_i_), and cell capacitance (Cm)).
  c. Repeat the previous step to gather more data per cell.
    ***Note:*** We recommend recording only one cell per MG-ALI-CO, as this allows for pairing of the patch-clamp recording to the analysis of the biocytin-filled cell. This enables correlations of cell morphology and electrophysiological properties.
  d. Label a 2 mL Eppendorf tube and transfer the MG-ALI-CO by gently lifting the membrane using tweezers.
  e. Fix the MG-ALI-CO as previously described in step 1 and 2 of “2D immunohistochemistry of MG-ALI-CO”.
24. Select cells to analyze the obtained electrophysiological recordings.
  a. Include cells with the following criteria: Rs < 25 MΩ, I_leak_ > − 150 pA and Vm < − 40 mV.

**CRITICAL:** It is important to adhere to the cut-off parameters to ensure the quality of the recording data.

25. Convert the obtained electrophysiological recordings .dat files to .abf format, open the .abf files in Clampfit.

***Note:*** Python script available at GitHub repository can be used to convert .dat files to .abf format (see key resources table). Alternatively, analysis software of choice can be used to directly open and analyze .dat files.

26. Assess the passive membrane properties of the cell in Clampfit.
  a. Calculate R_s_ as ratio of the test pulse amplitude to the amplitude of the recorded capacitive current.
  b. Determine R_i_ by dividing the test pulse amplitude by the amplitude of the steady-state current measured at the end of the test pulse.
  c. Calculate Cm by dividing the total charge (Q) by the test pulse amplitude, where Q is obtained by calculating the area under the capacitive current curve from the onset of the test pulse to the point at which the steady-state current is reached (Taylor, 2012^21^).
  d. Exclude recordings from analysis if the average R_s_ > 25 MΩ or the R_s_ changed > 30% during the recording.
  e. Also exclude cells when their resting membrane potential is > −40 mV.
27. Determine sEPSCs frequency and amplitude of included cells.
  ***Note:*** Individual sEPSC events are detected semi-automatically with the “Template search” function under “Event Detection”.
  a. Use a single representative sEPSC template for all recordings with “Event Polarity” selected as negative-going.
  b. Set the baseline as “In template”, and the “Template match threshold” to 3.5.
  c. Confirm or reject each identified event manually.
  d. Determine the frequency (Hz) by dividing the number of confirmed events by the total recording time (s) analyzed.
  e. Determine the amplitude (pA) by taking the average of all confirmed events.
  f. Identify and exclude outliers, for example by using the built-in “Identify outliers” analysis (ROUT, Q = 1%) in Prism 9.

## Expected outcomes

Recent advances in neural organoid technology enable the differentiation and culture of microglia in a physiologically relevant, brain-like 3D environment. The MG-ALI-CO system we present supports long-term survival of microglial cells and promotes a highly ramified morphology. We hypothesize that these parameters indicate reduced cellular stress due to higher availability of nutrients and oxygen and diminished necrotic core formation, which consequently favors a more homeostatic microglial profile.

This protocol describes 3 different experimental procedures that can be used for the analysis of MG-ALI-COs (or ALI-COs). Neuronal differentiation and fate acquisition are key markers of ALI-CO quality. Therefore, time points should be selected that allow assessment of relevant markers of these processes (see Gordon et al., 2021^22^ for more information on time points and markers usage). The ALI-CO model used here is cortical in origin and FOXG1-positive neuronal progenitors are expected to be present, even at later stages of development (Figure 3B). Deep-layer (CTIP2+) and upper-layer (SATB2+) cortical neurons are expected to be widely distributed in DIV140 ALI-COs (Figure 3C). It should be noted that long-term culturing eventually results in loss of rosette-like structures that characterize neural organoids. 2D IHC can also be used to visualize pre-synaptic (SYP) and post-synaptic (PSD-95) markers, as well as their co-localization (Figure 3D). The presence of synaptic markers, specifically the co-localization of pre- and post-synaptic proteins, indicates neuronal maturity and is expected to increase as culture time progresses. Of note, 3D IHC (Figure 4) offers the possibility to assess and quantify synaptic protein engulfment by microglia, as it allows for imaging of the entire microglial cell. Subsequently, the cell can be visualized in 3D, permitting visualization and quantification of synaptic proteins present in the microglia. This contrasts with 2D IHC, which often only captures part of the cell due to cryosectioning. However, both 2D and 3D IHC methods have their own advantages, as outlined in this protocol.

Microglia morphology as well as marker expression can be assessed by 2D IHC (Figures 3E and 3F). However, complex, whole-cell morphologies (*e.g.,* fine branching) are optimally visualized using 3D IHC (Figure 4C). Additionally, 3D IHC is required to accurately assess microglia distribution throughout the MG-ALI-CO (Figures 4D and 4E). Neuron sparse-labeling by biocytin filling followed by 3D IHC can reveal neuron-microglia interactions (Figure 6E). Electrophysiological patch-clamp recordings enable the quantification of sEPSC frequency and amplitude from MG-ALI-CO neurons (Figures 6C and 6D), which provides researchers with a guide to compare different conditions (*e.g.,* distinct iPSC lines).

Our MG-ALI-CO model provides new opportunities for investigating neuro-immune interactions in a human system where microglia number or genetic background can be tightly controlled. For example, it is possible to investigate cell autonomous vs. non-cell autonomous effects by using MG-ALI-COs with different genetic backgrounds (*e.g.,* diseased vs. healthy) with microglia derived from the same iPSC lines. In addition, the high level of neuronal maturity displayed by ALI-COs and possibility of long co-cultures allows the investigation of phenotypes that in other (short-lived) organoid systems are difficult to observe, *i.e.,* microglia effects on neuronal excitability or synapse pruning.

## Limitations

The MG-ALI-CO system requires a long culturing period. Long-term cultures often require a high workload and are more vulnerable to infections. In addition, only later time points can be investigated with this system, since the ALI-COs need to recover from the slicing procedure.

Another limitation of this model is related to MacPre generation. Although YS-EBs are formed by all iPSC lines, not all produce MacPre. This can be determined by examining the culture plates using a bright-field microscope and is also reflected in low absolute numbers of MacPre during the cell count after collection.

During the postprocessing of synapse IHC, it might be necessary to adjust the PSD-95 signal per image to obtain the best result. However, for PSD-95-related quantitative comparison between conditions, this adjustment needs to be consistent. Therefore, these analyses must be carefully performed to ensure accurate results, which might require image analysis expertise.

## Troubleshooting

### Problem 1

Low numbers of microglia in MG-ALI-CO 6 to 8 weeks after integration.

### Potential solution

Microglia integration efficiency can be assessed 2 weeks after integration by performing 3D IHC for IBA1. Cell density may vary depending on ALI-CO size or ALI-CO region (Figure 4E), but a range between 50–150 cells/mm^2^ is expected. Ensure that the following steps are followed to prevent low microglia numbers: only employ MacPre with a viability above 75%, perform MacPre collections up to and no later than week 5; prevent the MacPre-containing drop from overflowing (which can cause MacPre distributing mainly outside of the ALI-CO); freshly add IL-34 and M-CSF to the medium; and aim for optimal ALI-CO quality (*e.g.,* absence of necrotic parts, presence of expected neuronal cell types at the time point of interest).

### Problem 2

Microglia marker expression is highly variable (*e.g.,* between iPSC lines) and difficult to quantify from microscopy images.

### Potential solution

Sort out microglial cells from the MG-ALI-CO using a MACS-based approach to isolate CD11b-expressing cells (see Ormel et al., 2019^6^), and assess markers with for example RT-qPCR, RNA sequencing or Western Blot. Pool a minimum of 10 MG-ALI-COs to get sufficient levels of RNA or protein.

### Problem 3

The sectioning of the MG-ALI-CO in the cryostat leads to tissue curling.

### Potential solution

Often when tissue sections curl during cryosectioning, temperature settings are not optimal. Usually, it means that the object temperature is too low, therefore adjusting the temperature settings to a slightly higher temperature should help.

### Problem 4

It is difficult to target specific neuronal subtypes while patching.

### Potential solution

Utilize viral methods to induce cell-type specific expression of fluorescent markers. The fluorescent signals can be used as a guide during patching.

### Problem 5

The size of the MG-ALI-COs will expand over time, thereby hindering bright-field visualization during patching.

### Potential solution

Only patch cells around 500 μm of the edge of the MG-ALI-CO, where fewer cell layers and more mature neurons are typically found.

### Problem 6

Technical variability in MG-ALI-CO electrophysiological recordings.

### Potential solution

Many factors contribute to technical variability in patch-clamp electrophysiology experiments (*e.g.,* pipette properties, recording set-up stability and slight variations in the aCSF solution^23^). To prevent technical variation from impacting your results, perform recordings of all experimental conditions (*e.g.,* different iPSC lines or treatment) on the same day.

## Resource availability

### Lead contact

Further information and requests for resources and reagents should be directed to and will be fulfilled by the lead contact, R. Jeroen Pasterkamp (r.j.pasterkamp@umcutrecht.nl).

### Technical contact

Questions about the technical specifics of performing the protocol should be directed to the technical contact, R. Jeroen Pasterkamp (r.j.pasterkamp@umcutrecht.nl).

### Materials availability

This study did not generate new unique materials or reagents.

### Data and code availability

This study did not generate/analyze datasets/code.

## Acknowledgments

We thank Youri Adolfs for technical support with microscopy data acquisition and the MIND facility (mindresearchfacility.nl). This work was supported by Stichting ALS Nederland (TOTALS, ALS-on-a-Chip, ATAXALS, MUSALS, and GoALS); the MAXOMOD and INTEGRALS consortia (E-Rare-3, the ERANet for Research on Rare Diseases); the ALS CURE Project;, Alzheimer Nederland; the EU Joint Program Neurodegenerative Diseases (JPND; TRIAGE) (to R.J.P.); and the BRAINS Initiative of Alzheimer Nederland, EpilepsieNL, Hersenstichting and Stichting MS Research (GLIODEM), Alzheimer Nederland (ASTRODEM), and ZonMW (MODEM) (to E.M.H.).

## Author contributions

M.C.L., M.M., C.E.J., and R.J.P. conceived the project; M.C.L., M.M., C.E.J., C.F.M.H., and A.E. designed, performed, and analyzed the experimental protocol; M.C.L., M.M., C.E.J., and R.J.P. wrote the protocol and prepared figures; E.M.H. and R.J.P. acquired the funding; R.J.P. supervised the study.

## Declaration of interests

The authors declare no competing interests.
